# Future therapeutic strategies for olfactory disorders: electrical stimulation, stem cell therapy, and transplantation of olfactory epithelium—an overview

**DOI:** 10.1007/s00106-022-01249-8

**Published:** 2023-02-03

**Authors:** N. Gunder, P. Dörig, M. Witt, A. Welge-Lüssen, S. Menzel, T. Hummel

**Affiliations:** 1 Klinik und Poliklinik für Hals-, Nasen- und Ohrenheilkunde, Universitätsklinikum Carl Gustav Carus an der Technischen Universität Dresden, Fetscherstr. 74, 01307 Dresden, Germany; 2 Universitäts-HNO Klinik Basel, Basel, Switzerland; 3 Institut für Anatomie, Universitätsmedizin Rostock, Rostock, Germany

**Keywords:** Anosmia, Nose, Smell, Treatment, Implant, Graft

## Abstract

Olfactory disorders may be temporary or permanent and can have various causes. Currently, many COVID-19 patients report a reduced or complete loss of olfactory function. A wide range of treatment options have been investigated in the past, such as olfactory training, acupuncture, medical therapy, transcranial magnetic stimulation, or surgical excision of olfactory epithelium, e.g., in severe qualitative smell disorders. The development of a bioelectric nose, e.g., in connection with direct electrical stimulation or transplantation of olfactory epithelium or stem cells, represent treatment options of the future. The basis of these developments and the state of knowledge is discussed in the following work.

Temporary or persistent olfactory disorders can have various causes. Recently, a large number of patients have reported absent or diminished olfaction in the context of COVID-19 infections [62]. Although the prognosis for patients with prolonged postviral olfactory disorders can be considered rather good with a spontaneous remission rate of about 30% within one year. There are other causes such as olfactory disorders after traumatic brain injury, in which the prognosis regarding recovery of olfaction is comparatively poor [19].

Olfactory impairment is found in approximately one fifth of the population [8, 74]. Overall, approximately 5% are anosmic [38]. For affected individuals, olfactory impairment represents a significant reduction in quality of life and they often suffer from depression [17, 56]. Regardless of the cause, most patients hope for a rapid and, above all, complete remission of their olfactory function [6]. In the past, many treatment options have been investigated, ranging from olfactory training [57, 67], acupuncture [18], and drug therapies [32, 58, 63] to transcranial magnetic stimulation [29] or, for example, surgical resection of the olfactory mucosa in cases of pronounced qualitative olfactory disorders [39, 46]. The development of a bioelectric nose, for example, in combination with direct electrical stimulation of the olfactory bulb (OB), and the transplantation of olfactory mucosa or stem cells represent advanced treatment options.

## Basic principles

Understanding the physiology of smell is crucial for treatment approaches. Olfactory impressions arise when odor molecules bind to olfactory receptors (OR) in the cilia of olfactory receptor neurons (ORN) of the olfactory mucosa. A transduction cascade is then activated, leading to depolarization of the ORN and consequently resulting in an action potential activating the OB from where the signal is relayed to other brain areas [9, 78]. Odor perception is significantly influenced by experience and physical states such as the sensation of hunger. Conversely, odors also influence memories and emotions [35].

Humans possess approximately 370 OR-encoding genes [25, 75]. Only one specific receptor type is encoded in each ORN [65, 73]. The respective ORNs possessing the same OR project with their axons to only a few topographically defined glomeruli [44, 60, 72], this is called “convergence,” which is very pronounced in the olfactory system compared to other sensory systems. Thus, a concrete activation pattern is created in the OB [70], based on which the brain is able to identify the corresponding odor qualities. It is assumed that this sensory map is identical in different individuals [41].

However, in addition to ORNs, the olfactory mucosa also contains basal cells, which in turn are precursor cells for ORNs, supporting cells, and microvillar cells. Basal cells can be differentiated into horizontal and globose basal cells (GBCs; [64]). Due to its mitotic activity and differentiation ability, the olfactory mucosa has a lifelong regenerative capacity, which is unique for a human sensory epithelium. The task of the microvillar cells is not yet clear, but they also may serve sensory functions. Supporting cells maintain the ionic balance, among other functions. In the context of COVID-19-associated olfactory disorders, they appear to play a central role [12]. The lamina propria of the olfactory mucosa also contains olfactory ensheathing cells, a type of glial cell that surrounds ORNs. They provide electrical insulation and extend from the periphery, after passing through the approximately 1–2-mm openings of the lamina cribrosa, to the central nervous system up to the OB [16, 66, 79].

Old age, infections, chronic sinonasal diseases, craniocerebral trauma, or neurodegenerative diseases represent the most common causes of olfactory impairment [20]. As a consequence, olfaction may be completely absent, a condition that is called “anosmia.” Depending on the cause of the olfactory disorder, the symptoms may be temporary or constant. With regard to COVID-19-associated olfactory disorders, it is noteworthy that significant improvement often occurs two to four weeks [7]. In addition, it was noted relatively early in the pandemic that olfactory impairment is an early symptom and this is typically noticed around the fourth day of illness. The sudden onset of olfactory dysfunction is more typical of postinfectious olfactory dysfunction [52]. Since SARS-CoV‑2 patients rarely complain of additional rhinitic symptoms, pathophysiological considerations suggest that the olfactory dysfunction is not primarily a conductive problem, but instead one of damage to the olfactory mucosa or a neuronal affection [33]. This assumption supports the knowledge gained in the meantime that SARS-CoV‑2 enters the cell via the surface receptor angiotensin-converting enzyme (ACE) 2, which is mainly expressed by the supporting cells. This is a possible explanation for the rapid improvement in olfactory function in many cases, since the ORNs themselves are only indirectly affected [12]. Furthermore, a possible entry of the virus via neuropilin 1 into the brain has been discussed, such that COVID-19-associated olfactory dysfunction could also result from an impairment of the processing centers in the brain [33]. Using SARS-CoV‑2 as an example, it is evident that the cause of olfactory dysfunction also influences the improvement of olfactory function during progression [77]. Despite various efforts to develop an effective therapy for olfactory disorders, success in this field is limited. When a person loses an entire sensory quality, this represents a significant impairment for daily life. In terms of hearing, congenital as well as acquired profound hearing loss, bordering on deafness, can often be treated relatively well with the help of a cochlear implant [47, 55]. An analogy for blind individuals is the retinal implant for the treatment of degenerative retinal diseases. The first implant was approved in the United States in 2013 [59]. These examples show that electrical stimulation of sensory organs or sensory nerves can lead to sensations that can be of extraordinary help for orientation in daily life.

In addition to electrical stimulation, which has been shown to be quite successful in restoration of other senses, another promising therapeutic approach may be the functional restoration of the olfactory mucosa. The idea of replacing defective organ systems with healthy ones is not novel. Skin transplants were described in Asia as early as 600 B.C. [2]. The first successful kidney transplantation was performed in Paris in 1953 [28]. The example of hemato-oncological patients can also be used to demonstrate the importance of stem cell transplantation in our current therapeutic concepts [4]. Such therapeutic approaches are particularly useful in the treatment of olfactory disorders, since stem cells are naturally present in the healthy olfactory mucosa and the olfactory mucosa regenerates from them.

The present work is intended to provide an overview of the topic of electrical stimulation of the olfactory system and the feasibility of a bioelectric nose. In addition, current scientific findings regarding a possible transplantation of olfactory mucosa as well as stem cell therapy to restore olfactory function are discussed.

## Electrical stimulation of the olfactory system

### Electrical olfactory activation in animals

As early as 1959, Ottoson published a study on electrical stimulation of the nasal mucosa in frogs. Through this, electrical potential changes of the olfactory nerve and OB could be triggered. Different areas of the mucosa were stimulated but only stimulation near the olfactory mucosa led to a potential change in the OB [53]. This study fundamentally demonstrated that electrical stimulation of the olfactory nerve and OB are feasible.

Coelho and Constanzo performed studies using a 32-channel electrode for spatial mapping of the OB in rats. For stimulation, they used natural scents and direct electrical stimulation. They recorded the responses to each stimulus in different areas of the OB. With the help of different programs, patterns could be generated for the natural odorants, which represent the corresponding neuronal activities for the respective odor qualities. Thus, as in previous studies, the authors were able to demonstrate that different odors lead to localized activation patterns in the OB. Localized responses could also be derived for direct electrical stimulation depending on the localization of the stimulus [14].

To understand the neuronal activity of the ORN after presentation of an olfactory or possibly electrical stimulus, Dong and colleagues implanted a self-made 16-channel microelectrode in the OB in rats. Five different scents (anisole, citral, carvone, isobutanol, isoamyl acetate) led to different neuronal activities of the ORN and varying activation patterns could be displayed depending on the stimulation with the different scents. A decoding algorithm was used to identify the odorants [22]. Based on this work, the feasibility of discriminating odor qualities by a bioelectric nose was ultimately demonstrated. However, difficulties arose in the discrimination of odor mixtures as well as different odor concentrations.

### Electrical stimulation of the human olfactory mucosa

The first studies on the stimulation of the olfactory mucosa in humans were published as early as the 19th century. In these studies, odor sensations were generated by electrical stimulation via an endonasally placed electrode. For example, subjects described the perceived odor as a “lighted match” [3].

**Fig. 1:**
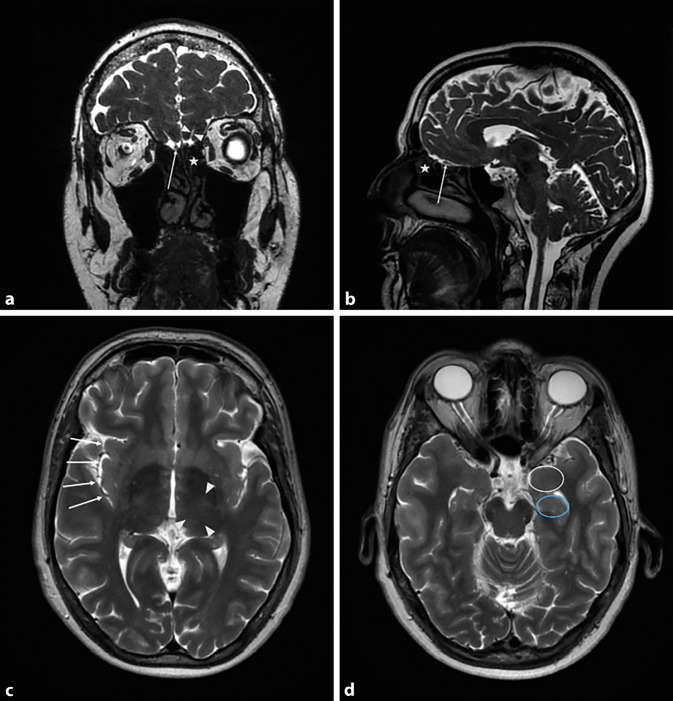
**a** Magnetic resonance imaging (MRI) of the skull; coronal T2-weighted image. Olfactory bulb (*arrow*), olfactory sulcus (*triangles*), olfactory mucosa (*star*). **b** MRI of the skull; sagittal T2-weighted image. Olfactory bulb (*arrow*), olfactory mucosa (*star*). **c** MRI of the skull; axial T2-weighted image. Insula (*arrows*), thalamus (*triangles*). **d** MRI of the skull; axial T2-weighted image. Amygdala (*white circle*), hippocampus (*blue circle*)

In 1973, Uziel published the results of his experiments in which a total of 21 participants had placed two different electrodes, a silver as well as a coated silver–silver chloride–sodium chloride (Ag-AgCl-NaCl) electrode, in the area of the olfactory mucosa (Fig. 1a, b) under visual control. During anodal stimulation, Uziel observed the emergence of olfactory sensations defined by threshold, latency, duration, and quality. Odor quality was described as “almond” by five normosmic subjects, and three others reported a “burnt” odor sensation. Other olfactory impressions were “purulent” and vanilla. By contrast, only trigeminal sensations such as “burning” and “stinging” could be elicited by cathodic stimulation and the Ag-AgCl-NaCl electrode. However, the observations of anodic stimulation were attributed to the release of chlorine, which may have triggered the olfactory sensation [71].

In a study by Straschill and colleagues, electrical stimulation of the olfactory mucosa resulted in suppression of the perception of odors. Furthermore, when an electrical stimulus was applied at a specific interval after an odor stimulus, it was observed to lead to a similar odor perception as the previously presented scent. Electrical stimuli without a preceding scent stimulus also resulted in “cacosmic” perception in three patients; these were patients with temporal lobe epilepsy and olfactory auras [68].

Ishimaru et al. first recorded EEG-dependent evoked potentials after electrical stimulation of the human olfactory mucosa in 1997. In five individuals, the olfactory mucosa was electrically stimulated unilaterally via a bipolar Ag electrode. Simultaneously, brain waves were recorded with two electrodes (one frontal and one lateral). Although potentials could be registered after electrical stimulation, no olfactory sensation was reported. Stimuli outside the olfactory cleft elicited a sensation of pain, most likely due to an affliction of the trigeminal nerve [34].

In 2016, Weiss and colleagues assessed different experimental setups with 60 volunteers, applying electrical stimuli via an intranasal Ag electrode. In the first experimental arrangement, the hypothesis that stimulation in the area of the olfactory mucosa leads to odor perception could not be confirmed based on stimulation of different areas of the nose (middle and upper conchae, dorsal edge of the septum and olfactory cleft). At no time did the volunteers report an odor perception. However, simultaneous electrical stimulation in the area of the olfactory mucosa resulted in modulation of an olfactory impression. Pleasant odors (rose and chocolate) were perceived as significantly less pleasant by simultaneous electrical stimulation. In addition, 20 participants underwent functional magnetic resonance imaging (MRI) to illustrate the effect of electrical stimulation of the olfactory mucosa. This revealed a neuronal response specifically in the primary olfactory cortex, indicating the correct location of the electrode in the olfactory cleft, yet no olfactory perception could be induced by electrical stimulation of the olfactory mucosa [76].

In summary, the majority of these studies demonstrate that direct electrical stimulation of the olfactory mucosa can lead to an olfactory perception. Furthermore, electrical stimulation in the area of the olfactory cleft leads to modulation of the presented odors. The perception of these can be suppressed, but also subsequently evoked.

### Electrical stimulation of the OB in humans

In studies by Penfield and Jasper, direct electrical intraoperative stimulation of the OB (Fig. 1a, b) on awake patients resulted in a perception of rather unpleasant odors such as burnt rubber or manure [54]. However, stimulation of the OB can also be achieved by placing electrodes in the area of the lateral part of cribriform plate after performing sinus surgery. Electrical stimulation resulted in subjective odor perception in three out of five individuals. These were described as onion-like or antiseptic and sour or fruity and bad. Thus, transethmoidal stimulation of the OB represents a novel approach in the development of the bioelectric nose [31].

### Electrical stimulation of the cortex and other brain areas in humans

#### Orbitofrontal cortex.

Kumar and colleagues published a study in which they implanted subdural electrodes in the right and/or left frontal, temporal, parietal, and occipital areas of 16 children with focal epilepsy. Electrical stimulations in the ventral area of the medial frontal lobe led to olfactory perception in 11 children. This odor sensation was mostly described as unpleasant, smoke, or garbage. Two children reported pleasant smells such as strawberries or good food. Electrical stimulation of other brain areas did not result in olfactory sensations. Olfactory “hallucinations” could only be produced by stimulations near the OB or the olfactory tract [36].

In contrast to what has been described previously, Bérard et al. succeeded in producing pleasant olfactory impressions by electrostimulation of the medial orbitofrontal cortex. In this study, electrical pulses were applied via variously localized depth electrodes in eight patients with temporal lobe epilepsy. After stimulation in the olfactory sulcus, the medial orbital sulcus or the medial orbitofrontal gyrus, five of the eight individuals reported pleasant olfactory sensations. For example, these were described as lemon, coffee, perfume, eucalyptus, and fruit or vegetable. A change in stimulation amplitude resulted in a change in odor perception in three of the five patients [5].

#### Insular cortex.

On the basis of retrospective trials of electrical stimulation of the insula (Fig. 1c), it was mapped in terms of elicited olfactory and gustatory sensations. A total of 651 studies were evaluated in which the insula of 221 patients with drug-refractory epilepsy was stimulated via a stereotactically implanted depth electrode. Six times, an olfactory sensation was reported, which was triggered by electrical stimulations in the area of the middle dorsal insula. Furthermore, the results showed that there is a spatial overlap between gustatory, olfactory, and oral somatosensory representations in this area [42].

#### Amygdala and hippocampus.

In 1967, Andy published a case report of a patient who reported the perception of foul odor after electrical stimulation of the right amygdala (Fig. 1d). However, stimulation of the hippocampus (Fig. 1d) did not produce an odor sensation [1].

#### Thalamus.

Electrostimulation of the thalamus (Fig. 1c) can also induce olfactory hallucinations. In Nashold and Wilson’s studies, unpleasant odor perceptions could be produced in three of five individuals (burnt, burnt rubber, or chloroform). One participant perceived pleasant odors such as hay and clove. Furthermore, one participant reported a vague odor sensation that reminded him of “South Dakota” [50].

Thus, the studies suggest that electrical stimulation can lead to different and specific odor sensations depending on stimulation parameters and location of the stimulus. A summary of the applications sites and their results are shown in Table 1.

**Table 1:** Overview of electrical stimulation on the olfactory system

Source (year)	Study population	Methods	Results
*Electrical olfactory activation in animals*			
Ottoson (1959) [53]	Frogs	eStim nasal mucosal stimulation	In the area of the olfactory mucosa → potential changes in OB
Coelho and Costanzo (2016) [14]	8 Rats	Olfactory stimuli recording of neuronal activity in the OB (*n* = 8) eStim of the OB (*n* = 4)	Localized activation patterns in the OB by different odor stimuli eStim at different localizations of the OB → localized activation of the OB using evoked potentials
Dong et al. (2013) [22]	44 Rats	eStim by 16-channel micro-electrode at the OB Presentation of five different odors	Derivation of odor-dependent activation patterns of the ORN Scent identification by decoding algorithm of the activation patterns (*n* = 4)
*Electrical stimulation of the human olfactory mucosa*			
Aronsohn (1886) [3]	4 Healthy individuals	Bilaterally endonasally placed electrode	Odor perception “match” (*n* = 2)
Uziel (1973) [71]	21 (Healthy individuals and ENT patients)	eStim with various electrodes (Ag and Ag-AgCl-NaCl)	Anodal stimulation → odor perception “almond” (*n* = 5), “burnt” (*n* = 3), vanilla (*n* = 1) and “purulent” (*n* = 1). Ag-AgCl-NaCl: no olfactory sensation, but sensation of pain
Straschill et al. (1983) [68]	10 Healthy individuals, 5 patients with epilepsy	eStim and presentation of olfactory stimuli	Suppression of olfactory sensation Cacosmic olfactory impressions without odor stimulus (*n* = 3)
Ishimaru et al. (1997) [34]	5 Healthy individuals	eStim via Ag electrode	No generation of olfactory sensations
Weiss et al. (2016) [76]	60 Healthy individuals	eStim of different nasal sections fMRI (*n* = 20)	Modulation of presented olfactory stimuli only in eStim of olfactory mucosa. No generation of olfactory sensations by eStim
*Electrical stimulation of the OB in humans*			
Penfield and Jasper (1954) [54]	5 Patients with epilepsy	Intraoperative eStim of the OB in the awake patient	Unpleasant odor sensation
Holbrook et al. (2019) [31]	5 Patients with CRS	Transethmoidal eStim of the OB	Odor perceptions (*n* = 3): onion-like, antiseptic, sour, fruity, bad
*Electrical stimulation of the cortex and other brain areas in humans*			
Kumar et al. (2012) [36]	16 Children with epilepsy	eStim via subdural electrodes	Stimulation near OB or olfactory tract → olfactory sensations (*n* = 13)
Bérard et al. (2020) [5]	8 Individuals with epilepsy	eStim via deep brain electrode	Stimulation at medial orbitofrontal cortex → olfactory perception (*n* = 5)
Mazzola et al. (2017) [42]	221 Individuals with epilepsy	eStim of the insula via deep brain electrodes	Olfactory sensations from stimuli at mediodorsal insula
Andy (1967) [1]	1 Individual with epilepsy	eStim amygdala and hippocampus (right-sided)	Electrostimulation of amygdala → olfactory sensation (foul), electrostimulation of hippocampus without olfactory sensation
Nashold and Wilson (1970) [50]	5 Individuals with neurological disease	eStim via depth electrodes on the awake patient	Electrostimulation of the thalamus → various olfactory impressions (rubber, smoky/burnt, chloroform, clove)

*Ag* silver, *Ag-AgCl-NaCl* silver–silver chloride–sodium chloride, *CRS* chronic rhinosinusitis, *eStim* electrical stimulation, *fMRI* functional magnetic resonance imaging, *n* number, *OB* olfactory bulb

## Neurogenesis in the olfactory mucosa

Other therapeutic efforts focus on the direct functional restoration of the damaged olfactory mucosa. The ORN play an important role, which are responsible for both the reception of odors and signal transduction. Consequently, knowledge of the regeneration and neurogenesis of these cells is central to the development and understanding of such treatment approaches.

As early as the 19th century, concepts existed for characterizing cells in vertebrates as a function of their regenerative capacity. With respect to neurons, it was assumed at that time that they display stable cell behavior and accordingly cannot be replaced, even if they are destroyed. Due to the lack of studies on the olfactory mucosa, this idea persisted for a relatively long time. In the late 1970s, however, the understanding changed fundamentally. Using radiolabeled ^3^H‑thymidine, a thymidine analog, it was shown that divisible progenitor cells differentiate into ORN, providing a lifelong neurogenetic matrix in vertebrates. After nerve transection of ORN, nerve cell regeneration was also observed. As part of regeneration, axon sprouting toward the forebrain even occurred when the OB had been removed from the mice [26].

Using nasal mucosal biopsies taken during autopsies as well as nasal surgery, Murrell et al. investigated the neurogenesis of the olfactory mucosa in humans. For this purpose, they established serum-free cell cultures and stimulated them with fibroblast growth factor 2 (FGF-2). Within the first 15–20 days of stimulation, there was a remarkable increase in bipolar cells that were immunohistochemically positive for olfactory marker protein (OMP), which is typically found in mature ORN. Radioactively labeled thymidine was finally used to demonstrate differentiation into ORN in vitro. The age of the oldest individual from whom a biopsy was taken was 72 years, and thus the work demonstrated neurogenesis in the human olfactory mucosa into older age [49]. A recent paper confirms that neurogenesis occurs in the olfactory mucosa in humans into adulthood. The authors were able to demonstrate that various neurogenic cell stages were present in the individuals studied (age 41–52 years). However, in contrast to rodents, the proportion of immature receptor neurons was rather high at 55%, whereas in rodents this part accounts for only 5–15% [24]. However, the fact that the neuroregenerative capacity is not unrestricted throughout life, but that a certain fatigue can be assumed, was demonstrated in a transgenic mouse model. In the genetically modified animals, premature cell death of ORN occurred, which in turn led to increased cell turnover. After only two months, the olfactory mucosa resembled that of older humans with areas lacking neurons or GBCs, which are a prerequisite for neuronal regeneration. In these areas, tissue changes in the sense of respiratory metaplasia were also observed. As a consequence of this change, there is a reduced neuronal stimulation of the OB, which could also be seen in the reduced volume (shrinkage) of the glomeruli [13]. That olfactory deprivation is associated with a reduction in the volume of the OB was also shown many years ago by Meisami and colleagues [43]. Neurogenesis in the olfactory mucosa is dependent not only on age, but also on the olfactory exposure of an individual. In a study in mice, olfactory stimulation was found to result in upregulation of certain subtypes of ORN, suggesting an adaptive and plastic nature of the olfactory mucosa [40]. In addition, there appears to be a spatial determination within the olfactory mucosa with respect to the maturation of ORN. Coleman and colleagues removed progenitor cells from the dorsal region of the olfactory mucosa in mice and reinserted them further ventrally into the olfactory mucosa. They showed that after transfer, the majority of neurons typically expressed surface glycoproteins ventrally, but lost the expression of certain enzymes [15]. Thus, several factors are responsible for the regeneration and plasticity of the olfactory mucosa.

The olfactory mucosa has an enormous regenerative potential. This is influenced not only by age but also by various other factors. This plasticity of the mucosa could also be used in a therapeutic context. In the following, the therapeutic use of stem cells is presented and the possibility of transplantation of olfactory mucosa is discussed.

## Stem cell transplantation

The bone marrow contains a heterogeneous cell population with multipotent stem cells, progenitor cells, and differentiated cells. The high plasticity of bone marrow cells allows them to colonize different tissues and differentiate into diverse cell lineages. This potency makes bone marrow cells a good candidate for cell therapies [21].

In 2005, Tsujigiwa et al. showed that transplanted bone marrow cells can migrate into the olfactory mucosa of the recipient, grow there, and also differentiate into olfactory neurons. For this purpose, bone marrow cells from transgenic mice expressing green fluorescent protein (GFP) were transplanted into pre-irradiated mice. The GFP-positive cells were detected in the olfactory mucosa as early as two weeks after transplantation. After another 3.5 months, the cells were found not only in the basal layer of the olfactory mucosa, but also in the middle mucosal layer, where mature olfactory ORN are typically located. Immunohistochemical analyses for OMP also provided evidence that these were mature ORN [69].

Since the rate of newly formed ORN was rather low in the study by Tsujigiwa et al., another group wanted to optimize this by transplanting bone marrow stem cells only. Transfer of stem cells labeled by bromodeoxyuridine (BrdU) was performed intravenously in one group of mice and locally by transnasal injection in the other group. Immunohistochemically, cells in the olfactory mucosa were found to be positive for GAP43, which is typically expressed in GBCs, in both modes of application. At three weeks after transplantation, the cells had reached the middle layer of the olfactory mucosa. However, differentiation of the progenitor cells into mature ORN did not occur. The results of this study also suggested that the efficiencies of cell transplantation growth rate were comparable for local and systemic administration [51].

The use of stem cells has also been investigated in OB. A Spanish research group conducted therapeutic trials with bone marrow stem cells in transgenic mice showing loss of mitral cells in the bulb, which is the second neuron of the olfactory pathway. Loss of mitral cells in the OB leads to decreased odor detection and odor discrimination in the mice. Transplantation of bone marrow cells into the bulb resulted in the formation of microglial cells, but not mitral cells or other neuronal cells. Strikingly, the normal progressive loss of mitral cells was markedly reduced in the transplanted mice. Consequently, it can be suggested that microglial cells appear to have a protective effect with respect to mitral cells. It is already known from previous work that microglial cells can release neuroprotective substances. Through electrophysiological studies, it also became clear that the transplanted mice showed an improvement in odor recognition and odor discrimination compared to the control group [21].

Kurtenbach and colleagues pursued a new therapeutic approach to olfactory disorders by transplanting tissue-specific stem cells. For their study, they created a transgenic mouse model in which they removed the interflagellar transport protein from the DNA of the mice to induce hyposmia in the animals. The hyposmia was objectified by measuring markedly reduced electrophysiological responses to banana scent (amyl acetate) in the treated mice by electro-olfactography. As a histologic correlate, they found ciliopathy of the ORN. Regarding the transplanted stem cells, only olfactory mucosa stem cells that were c‑KIT surface receptor-positive were used. c‑KIT-positive GBCs have the capacity for neurogenesis. To generate as many of these stem cells as possible for transplantation, donor mice were treated with methimazole for two days prior to collection of the cells, resulting in cell death of mature ORN and concomitant upregulation of GBCs. The c‑KIT-positive GBCs were delivered into the hyposmic mice by nasal injection. At four weeks after transplantation, the olfactory mucosa was examined histologically: Cilium-bearing ORN were formed from the transplanted stem cells. Likewise, axon growth was detected in the olfactory nerve; axon sprouting even extended into the glomeruli of the bulb. Periglomerularly, tyrosine hydroxylase-positive cells were also found, indicating scent-induced cell activity. Furthermore, electro-olfactographic measurements and also behavioral tests showed better results of the cell-treated mice compared to the control group. Thus, the transplanted cells seem to not only grow into the olfactory mucosa and differentiate, but also lead to an improvement in olfactory function [37].

The potential of stem cell therapies in the use of olfactory disorders seems promising. One of the major challenges is certainly the safety aspect of such therapeutic approaches, especially with regard to uncontrolled cell development and cell migration with a corresponding risk of degeneration.

## Transplantation of olfactory mucosa

In addition to cell therapy, there are also efforts at transplantation of olfactory mucosa. As early as 1983, olfactory mucosa from neonatal rats was transplanted into the parietal cortex and fourth ventricle of adult and newborn animals. Neurogenesis persisted despite the ectopic location of the mucosa. Axon bundles could even be demonstrated to penetrate the recipient brain. However, there was no formation of olfactory glomeruli [45]. Holbrook and colleagues achieved similar results with grafts from postnatal mice. The graft survived in 85% of cases [30].

However, olfactory mucosa can be transplanted not only into the cortex but also into the OB. The survival rate of the graft in both locations was 83%. Characteristic of the transplanted olfactory mucosa was the appearance of vesicles in the vicinity of olfactory mucosal cells. Histological studies also revealed that the multilayered structure of the olfactory mucosa was preserved, and ciliary-bearing cells were also visualized on the epithelial surface, but functional synapses with the bulb were not [80].

Overall, it has been shown that olfactory mucosa can be transplanted into various sites of the cortex as well as into the fourth ventricle and the OB (Table 2). How their functionality relates to olfaction has not been further investigated in any study to date.

**Table 2:** Overview of stem cell and olfactory mucosa transplantation

Source (year)	Study population	Methods	Outcome
*Stem cell transplantation*			
Tsujigiwa et al. (2005) [69]	Mice	aTrans of GFP-positive bone marrow cells by i.v. injection	Labeled bone marrow cells migrate into the OM of the recipient and partially differentiate into ORN
Ochi et al. (2010) [51]	Mice	aTrans of BrdU-labeled bone marrow stem cells by i.v. or transnasal injection	Comparable efficiency of cell transplantation accrual rate in the OM regardless of the mode of application No maturation of progenitor cells in ORN
Diaz et al. (2012) [21]	Mice	aTrans of bone marrow stem cells by i.v. injection into transgenic mice with increased mitral cell loss in OB	Formation of numerous microglial cells in the OB with neuroprotective effect → reduced loss of mitral cells → improvement of odor recognition as well as odor discrimination
Kurtenbach et al. (2019) [37]	Mice	aTrans of stem cells of the OM into transgenic hyposmic recipients by means of nasal injection	Formation of cilium-bearing ORN incl. axon sprouting into the OB → improved olfactory function
*Transplantation of olfactory mucosa*			
Morrison et al. (1983) [45]	Rat	aTrans of neonatal OM into the parietal cortex and the fourth ventricle	Persistence of neurogenesis in the graft at ectopic location Formation of axons but not olfactory glomeruli
Holbrook et al. (2001) [30]	Mice	aTrans from postnatal OM into the parietal cortex	Graft survival in 85% with preservation of regenerative epithelial function
Yagi et al. (2009) [80]	Mice	aTrans from postnatal OM into the cerebellar cortex (*n* = 12) and OB (*n* = 6)	Graft survival at both sites at 83% Graft exhibits epithelial characteristics of normal OM

*aTrans* allogeneic transplantation, *GFP* green fluorescent protein, *ORN* olfactory receptor neurons, *OB* olfactory bulb, *OM* olfactory mucosa, *n* number

## Summary and outlook

In summary, on the one hand, clear indications for the feasibility of developing a bioelectric nose are revealed. Direct electrical stimulation of the OB may represent a therapeutic option for the treatment of anosmia if, for example, due to irreversible damage to the ORN, olfactory information cannot be transmitted to the OB to be finally processed in higher centers. However, it is still unclear up to which time point, i.e., up to which duration of the olfactory disturbance, electrical stimulation is promising. Humans are probably able to discriminate myriads of odors [11]. This shows that the demands on a potential olfactory implant are high. It must be able to detect the different olfactory information and transmit scent-specific electrical signals to the OB. However, it is known that the volume of the OB depends on age and olfactory function [10, 27, 48, 61]. It is unclear to what extent this aspect influences the development of an olfactory implant. For successful hearing and visual rehabilitation using a cochlear or retinal implant, an intact auditory or optic nerve is a fundamental requirement. Although most studies also refer to indirect or direct electrical stimulation of the olfactory nerve or OB, olfactory sensations could also be generated by subdural [36], cortical [42], and thalamic [50] stimulation. The electrical stimulations performed to date on humans partly required surgical interventions up to craniotomy, which is correspondingly invasive and associated with risks. Regarding the invasiveness, the use of stem cells would be preferable, which can be introduced relatively easily by nasal injection. However, the use of stem cells in olfactory disorders has not yet gone beyond animal models, although study results, especially those by Kurtenbach et al., showed very promising findings with regard to a possible functional restoration of the olfactory mucosa. However, when using stem cells, the carcinogenic potential of such a therapeutic approach would have to be considered. Furthermore, it is a matter of debate where the graft for transplantation of olfactory mucosa in humans could be obtained. In conclusion, both electrical stimulation and stem cell transplantation are promising methods for the treatment of olfactory dysfunction, but we believe that further research is needed in these areas to make their potential use in humans safe in the future.

## Declarations

For this article no studies with human participants or animals were performed by any of the authors. All studies mentioned were in accordance with the ethical standards indicated in each case.

The supplement containing this article is not sponsored by industry.
